# Hemostatic Analysis of Simulated *Gloydius ussuriensis* Envenomation Using Canine Blood: A Comparison of Thromboelastography and Classical Coagulation Tests

**DOI:** 10.3390/ani12030226

**Published:** 2022-01-18

**Authors:** Jong-Sun Lee, Jung-Hyun Kim

**Affiliations:** Department of Veterinary Internal Medicine, College of Veterinary Medicine, Konkuk University, Seoul 05029, Korea; n2984@konkuk.ac.kr

**Keywords:** venom-induced consumptive coagulopathy, snake envenomation, thromboelastography, in vitro study, veterinary sciences

## Abstract

**Simple Summary:**

Snake bites in companion animals are a major issue and cause life-threatening hemorrhage. Classical coagulation tests are performed to diagnose snake venom-induced coagulopathy; however, these tests often fail to detect early coagulopathy. This in vitro study using snake venom and canine blood aimed to explore the efficacy of thromboelastography, which enabled a comprehensive assessment of the coagulation process, compared with classical coagulation tests. Our results identified the usefulness of thromboelastography in detecting early venom-induced coagulopathy compared with classical coagulation tests. This finding may facilitate decision making in relation to the immediate initiation of anti-venom treatment or suspending unnecessary anti-venom administration, which often causes adverse reactions.

**Abstract:**

Snake envenomation may lead to venom-induced consumptive coagulopathy (VICC), usually diagnosed by classical coagulation tests (CCTs), such as prothrombin time (PT) and activated partial thromboplastin time (aPTT). However, the results of CCTs are frequently normal in the initial stages, which may delay anti-venom treatments. Thromboelastography (TEG) is a point-of-care and real-time diagnostic tool that enables a comprehensive assessment of the coagulation process. This in vitro study aimed to determine concentration-dependent changes in canine blood caused by *Gloydius ussuriensis* (*G. ussuriensis*) envenomation using TEG and CCTs. Lyophilized *G. ussuriensis* venom was reconstructed using mouse intravenous lethal dose 50 (LD50_iv_) and serially diluted to 25% LD50_iv_, 50% LD50_iv_, and 75% LD50_iv_ to reproduce VICC at different concentrations. Normal saline was used for the control. We compared TEG values of the reaction time (R), kinetic time (K), rate of clot formation (α-angle), maximum amplitude (MA), fibrinolysis at 30 min (LY30), and global strength of the clot (G) with those of PT, aPTT, fibrinogen, and platelet counts (PLTs). Most TEG parameters, except R and LY30, demonstrated statistically significant changes compared with the control at all concentrations. CCTs, except PLTs, revealed significant changes at ≥50% LD50_iv_. Thus, TEG could be a useful diagnostic strategy for early VICC and preventing treatment delay.

## 1. Introduction

Of the 3000 snake species worldwide, 30% are venomous [1]. According to the World Health Organization (WHO), 7400 snake bites are reported daily, thus resulting in an average of 300 casualties globally [2]. According to the big data availed by the Health Insurance Review & Assessment Service in South Korea, an average of 2580 venomous snake bites occur annually in South Korea [3]. However, there are no official reports on snake bites in veterinary medicine in South Korea. In North America, snake bites in dogs and cats are approximately 30-fold the number of human cases [4,5]. Contrarily, almost equal numbers of snake bites in humans and companion animals have been reported in Australia [6]. Taken together, the estimated number of snake bites in dogs and cats in South Korea is at least 2580 annually. This simple comparison may be subject to the statistical error; however, the gravity of snake bites in companion animals must not be neglected.

Four species of venomous snakes are found in South Korea, including *Gloydius ussuriensis, Gloydius brevicaudus,* and *Gloydius intermedius* of the Viperidae family and *Rhabdophis tigrinus* of the Colubridae family [7]. Snake venom is a bioactive mixture composed of several enzymatic and non-enzymatic proteins, peptides, and organic and inorganic compounds [8], which can induce neurotoxic, cytotoxic, myotoxic, and coagulopathic effects [9]. Furthermore, the venom composition may vary between genera and species in a particular family owing to different environmental factors, such as the prey, weather, and geographical location [10].

The coagulopathic effect is the most concerning aspect of the *Gloydius* species venom [11]. Venom injection through the fang into the bloodstream leads to the concurrent inhibition and depletion of coagulation factors, resulting in an increased bleeding tendency [12]. This phenomenon is termed venom-induced consumptive coagulopathy (VICC) because of its shared similarity with disseminated intravascular coagulopathy (DIC); however, it does not trigger severe organ damage or systemic thrombosis, which is common in DIC [13]. Animals with VICC usually exhibit pathological abnormalities, such as prolonged prothrombin time (PT), activated partial thromboplastin time (aPTT), low fibrinogen and antithrombin concentrations, elevated D-dimer levels, and thrombocytopenia [14,15,16,17]. However, laboratory results of classical coagulation tests (CCTs) are frequently normal in the initial stages and gradually become abnormal within a few hours to days [14,18,19]. This increases the need for novel tests to detect VICC during the aforementioned period.

Thromboelastography (TEG) is a point-of-care and real-time diagnostic tool that enables a comprehensive assessment of the coagulation process from clot generation to fibrinolysis [20]. Its usefulness ranges from transfusion in trauma patients to monitoring hemostasis during liver transplantation and cardiac surgeries [21,22]. In veterinary medicine, researchers have reported on the use of TEG during the diagnosis and treatment of VICC along with CCTs [14,15,16,17]. However, they have not compared the efficacy of TEG with that of CCTs.

The present in vitro study aimed to determine concentration-dependent changes in canine blood following envenomation with *Gloydius ussuriensis* (*G. ussuriensis*) venom using TEG and CCTs and to evaluate the sensitivity of each test for identifying results beyond the normal reference limits (NRLs).

## 2. Materials and Methods

### 2.1. Animals

This study was approved by the Institutional Animal Care and Use Committee of the Konkuk University (approval No. KU20217), Seoul, South Korea. All dogs were prospectively recruited at the Konkuk University Veterinary Medical Teaching Hospital from February 2021 to May 2021. We enrolled 10 healthy, client-owned dogs after acquiring informed consent from their owners. Before the experiment, all the dogs underwent a TEG test and were included in this study after normal results of the test were obtained for all parameters. To avoid the effects of external coagulation disturbances, we considered the following exclusion criteria: a history of anemia or bleeding disorder, a precedent use of steroids or anticoagulants in the past 30 days, a precedent use of non-steroidal anti-inflammatory drugs in the past 7 days, or a history of significant medical comorbidity.

### 2.2. Blood Collection

Four milliliters of blood were collected twice from each dog through jugular venipuncture in blood tubes containing 3.2% sodium citrate (1.8 mL, BD Vacutainer^®^; BD Vacutainer Systems, Plymouth, UK). All the blood samples were agitated on a roller to prevent premature clotting, followed by coagulation tests performed within 2 h of blood collection.

### 2.3. Preparation of Venom Solution and its Addition to Blood

**Figure 1:**
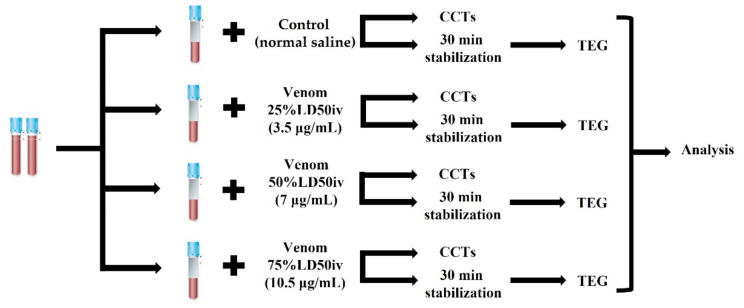
Timeline of the experimental design. The venoms of different concentrations were added to the blood samples in citrated tubes, whereas an equal amount of normal saline was added to the control blood sample. Each sample was assessed using CCTs and TEG, followed by statistical analyses. CCT, classical coagulation test; TEG, thromboelastography.

We stored the lyophilized venom of *G. ussuriensis* (Latoxan, Portes-lès-Valence, France) at −80 °C until the day of reconstitution with normal saline. The venom solution was aliquoted and re-stored at −20 °C to prevent the denaturation of the venom proteins and peptides. A standard point for measuring the extent of coagulation was set by using mouse intravenous lethal dose 50 (LD50_iv_, 1.139 mg/kg) to prepare venom solutions, according to the manufacturer’s guidelines. The calculations of 14 μg/mL were based on the estimated distribution of an 80 mL/kg blood volume in canines [23]. Thereafter, the venom solution was serially diluted to 25% LD50_iv_ (3.5 μg/mL), 50% LD50_iv_ (7 μg/mL), and 75% LD50_iv_ (10.5 μg/mL) to reproduce the venom-induced coagulation disorder using different concentrations. Next, we added a venom volume equivalent to 20% of the blood to the blood sample and an equal amount of normal saline to the control sample (Figure 1). Thereafter, we centrifuged the blood sample to perform CCTs (PT, aPTT, and fibrinogen) and prepared the slides for the manual platelet count. Simultaneously, blood samples for TEG were left for stabilization on the roller for 30 min [24,25]. CCTs were performed immediately after centrifugation. Subsequently, we carried out TEG after stabilization for 30 min, and the platelets were counted manually at the end.

### 2.4. TEG

We performed TEG coagulation assessment using a TEG^®^ 5000 Thromboelastograph^®^ Hemostasis Analyzer system (Haemonetics Corporation, Boston, MA, USA). The TEG machine comprised a holder with a cup maintained at 37 °C. The cup was filled with a 340 µL blood sample containing 1 mL citrated blood and 1% kaolin that were gently inverted five times and 20 μL of CaCl^2^ for recalcification to initiate the examination. A pin connected to the TEG machine by a torsion wire was positioned into the cup. Upon blood clotting, the resistance from rotational movement was sent to the torsion wire and converted into an electrical signal. A trace line was displayed on a computer monitor through the TEG program supplied by the manufacturer. We measured several parameters, including the reaction time (R), kinetic time (K), global values (G), α-angle, maximum amplitude (MA), and the percentage of fibrinolysis at 30 min following MA (LY30). R is the duration from the initiation of the assessment until the detection of clots showing a clot amplitude of 2 mm is reached. K is the duration from the end of R to a clot amplitude of 20 mm and represents a certain level of clot firmness. The α-angle denotes the tangent of a curve representing the clot formation rate. MA is the length of the widest amplitude at which the clot strength reaches its peak. G is the log derivation calculated from the MA value representing the global strength of the clot.

### 2.5. Classical Coagulation Tests

PT, aPTT, and fibrinogen were assessed using a coagulation analyzer CG02NV (A&T Corporation, Oshu, Japan). First, the reagent test card was inserted into the analyzer. Subsequently, a mixture of 25 μL of citrated plasma and a diluent (provided by the manufacturer) was applied to the test card using a pipette. Eventually, the results were displayed on a touch panel. PT and aPTT below the NRLs were considered normal. Platelet counts (PLTs) were determined manually under a microscope (OLYMPUS CX31RTSF, Olympus Corporation, Tokyo, Japan) in an oil-immersion field (OIF) to avoid the effects of platelet aggregation inducers isolated from the venom of the Viperidae family [26]. On visualizing the aggregated platelets in the OIF, each platelet was counted from the clumps, and the average values are presented.

### 2.6. Statistical Analyses

Statistical analyses were performed using Microsoft Excel (Microsoft Corporation, Redmond, WA, USA) and Prism 9.1.0 software (GraphPad Software, San Diego, CA, USA). Descriptive statistics are presented as interquartile ranges with the median, and differences between the 75th and 25th quartiles higher than 1.5-fold were defined as outliers. Data were tested for normality using the Shapiro–Wilk test. To determine the statistical differences between the control and venom concentrations evaluated with TEG and CCTs, we performed one-way repeated measure analysis of variance (RM-ANOVA) with pairwise comparisons to identify the difference between the groups. Statistical significance was set at *p* < 0.05. We obtained the NRLs for TEG parameters from a previous study [27], except for LY30 and G. Thus, G was calculated from MA based on a previously used equation as follows: *G* = (5000 × *A*) ÷ (100 − *A*) [28]. The sensitivities of detecting samples out of normal diagnostic ranges for TEG and CCTs were presented as a percentage and categorized into three groups with different sensitivities as follows: <70% indicated the low sensitivity; between 70% and 89% indicated the medium sensitivity; and >89% indicated the high sensitivity. Considering the absence of the NRL for LY30, we excluded the sensitivity and ANOVA results of LY30 from the analysis.

## 3. Results

### 3.1. Descriptive Analyses

**Figure 2:**
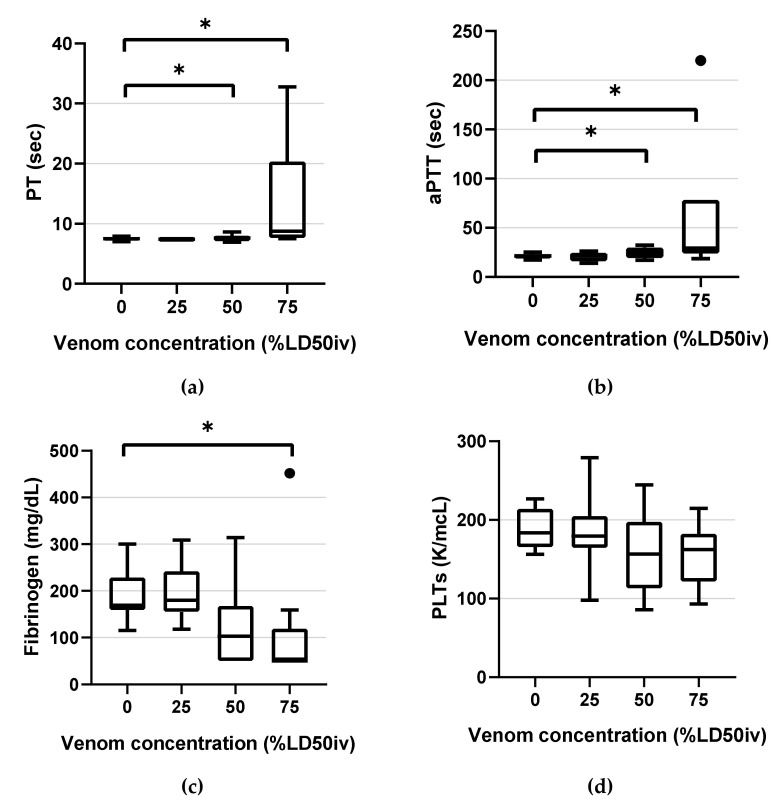
Box-and-whisker plots of the classical coagulation tests parameters: (**a**) PT (**b**) aPTT (**c**) fibrinogen (**d**) PLTs. Horizontal lines inside the boxes indicate the median. The upper boundary of the boxes indicates the top quartile, whereas the lower indicates the bottom quartile. An asterisk (*) indicates the venom concentration (% intravenous lethal dose 50 (LD50_iv_)) displaying a significant change compared with the control (*p* < 0.05). Differences between the 75th and 25th quartiles higher than 1.5-fold were considered as outliers and indicated by a black dot (•). PT, prothrombin time; aPTT, activated partial thromboplastin time; PLTs, platelet counts.

**Figure 3:**
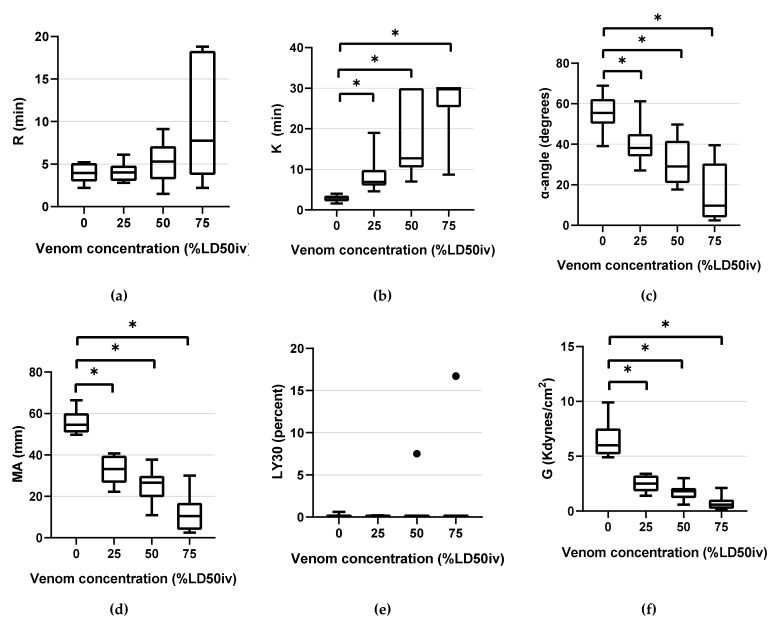
Box-and-whisker plots of TEG parameters: (**a**) R (**b**) K (**c**) α-angle (**d**) MA (**e**) LY30 (**f**) G. Horizontal lines inside the boxes indicate the median. The upper boundary of the boxes indicates the top quartile, whereas the lower boundary indicates the bottom quartile. An asterisk (*) indicates venom concentration (% LD50_iv_) displaying a significant change compared with the control (*p* < 0.05). Differences between the 75th and 25th quartiles higher than 1.5-fold are considered as outliers and indicated by a black dot (•). R, reaction time; K, kinetic time; α-angle, the rate of clot formation; MA, maximum amplitude; LY30, fibrinolysis at 30 min; and G, global strength of the clot.

Coagulation parameters of TEG and CCTs are illustrated in box-and-whisker plots (Figure 2 and Figure 3). The mean values of TEG and CCTs parameters are listed in Supplementary Tables S1 and S2. The data distribution assessment using the Shapiro–Wilk test revealed the following normally distributed data: R in the control and at 25% LD50_iv_ and 50% LD50_iv_; K in the control; α-angle in the control and at all concentrations; MA in the control and at all concentrations; G in the control and at 25% LD50_iv_ and 50% LD50_iv_; PT in the control and at 50% LD50_iv_; aPTT in the control and at 25% LD50_iv_ and 50% LD50_iv_; fibrinogen in the control and at 25% LD50_iv_; and PLTs in the control and at all concentrations. As the data were not normally distributed, and the sphericity was violated across the venom concentration, we used the non-parametric methods of RM-ANOVA and the Durbin–Conover test for pairwise comparisons. One value of LY30 was missing owing to the abrupt halt of TEG during the analysis for an unknown reason. None of the LY30 measures were normally distributed.

### 3.2. Concentration-Dependent Changes

#### 3.2.1. Classical Coagulation Tests

PT and aPTT demonstrated statistically significant changes compared with the control at 50% LD50_iv_ (*p* = 0.013, *p* < 0.001, respectively) and above (*p* < 0.001 and *p* < 0.001, respectively) (Figure 2). Fibrinogen displayed a statistically significant change only at 75% LD50_iv_ (*p* = 0.003; Figure 2). All PLTs were in the NRL and did not demonstrate significant changes at any concentration, compared with the control (Figure 2).

#### 3.2.2. TEG

**Figure 4:**
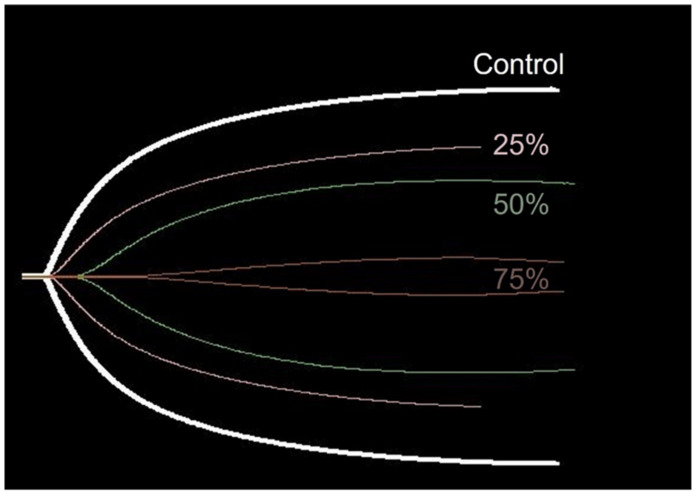
Typical TEG trace lines suggesting concentration-dependent changes from one of the dogs. The trace lines of the control and different venom concentrations (% LD50_iv_) are placed on the similar central axis for an easier comparison.

TEG parameters demonstrated statistically significant changes compared with the control, as the venom concentration increased. The following statistically significant changes were observed in the parameters at the respective concentrations: K (*p* < 0.001 at all concentrations), α-angle (*p* = 0.001 at 25% LD50_iv_, *p* < 0.001 at 50% LD50_iv_, and above *p* < 0.0001), MA (*p* < 0.0001 at all concentrations), and G (*p* < 0.0001 at 25% and 50% LD50_iv_, *p* = 0.002 at 75% LD50_iv_, Figure 3). However, R and LY30 did not exhibit statistically significant differences at any concentration (Figure 3). Figure 4 depicts typical TEG trace lines displaying concentration-dependent changes from one of the dogs.

### 3.3. Diagnostic Thresholds and Sensitivities

We determined the diagnostic thresholds (DHs) for mean values beyond the NRL. At 25% LD50_iv_, DHs were detected for K, MA, and G. At 50% LD50_iv_, we determined the DH for the α-angle, compared with those for R, PT, aPTT, and fibrinogen at 75% LD50_iv_. Moreover, we evaluated the sensitivities of each parameter, except for LY30 (Table 1). PT, PLTs, and R demonstrated a low sensitivity across the concentration range. Contrarily, aPTT and fibrinogen displayed low and medium sensitivities at concentrations of ≤50% LD50_iv_ and 75% LD50_iv_, respectively (Table 1). The α-angle displayed low, medium, and high sensitivities at 25% LD50_iv_, 50% LD50_iv_, and 75% LD50_iv_, respectively (Table 1). Meanwhile, K, MA, and G demonstrated a high sensitivity across the entire concentration range (Table 1).

## 4. Discussion

We intended to evaluate the efficacies of TEG versus CCTs in determining coagulation parameters following canine blood envenomation using simulated *G. ussuriensis* venom. Through the serial dilution of venom, we determined concentration-dependent changes and sensitivities. Our results were consistent with VICC caused by natural pit viper envenoming. However, in contrast to TEG and the rest of CCTs, PLTs did not demonstrate the coagulopathic effects of *G. ussuriensis* venom.

Conventionally, researchers perform coagulation tests such as PT, aPTT, 20 min whole blood clotting test, Lee–White clotting time, D-dimer, fibrinogen, fibrin degradation product (FDP), and PLTs, to diagnose VICC [12,13,14,15,16,17,29]. A previous report identified FDP as the most sensitive marker for detecting early VICC [30], whereas another confirmed PT and aPTT as useful diagnostic tools for VICC [31]. However, the aforementioned tests occasionally generated results within the NRL in envenomated patients, thereby indicating that CCTs may lead to false-negative results and/or delay in anti-venom treatments [32,33,34]. PT and aPTT are easily accessible in most laboratories and have the advantage of a short turnaround evaluation time of approximately 10 min. Nonetheless, certain characteristics of coagulation tests lead to limitations. For instance, the results of PT and aPTT are achieved within approximately 10 min before fibrin polymerization. In other words, these tests neither analyze the status of thrombin stability and fibrinolysis nor the circumstances lacking in protein C or antithrombin [12,35,36]. Since PT and aPTT evaluate the clotting time in the in vitro setting, they do not represent the in vivo interaction between coagulation factors and platelets [37]. Moreover, delays in PT and aPTT imply a specific coagulation factor deficiency during the coagulation cascade, thus necessitating further tests to specify the cause [37]. Similarly, fibrinogen, PLTs, and FDP produce only partial results at a specific point during coagulation, thereby warranting further tests [13]. Therefore, CCTs are unable to completely detect VICC characterized by platelet dysfunction and/or coagulation deficiencies.

The performance of TEG in the present experiment supposedly surpassed the ability of CCTs to detect VICC in the early stages. Of the TEG parameters, K, MA, and G displayed a high sensitivity even at the lowest venom concentration of 25% LD50_iv_, compared with the low sensitivity of the CCTs at 25% LD50_iv_. However, R displayed no significant changes at any concentration, and the sensitivity was low at all concentrations, thereby indicating its limited ability to detect coagulopathy caused by snake venom. Contrarily, the R-value tended to increase with the venom concentration (Figure 4). The experiment with 100% LD50_iv_ during the pilot study did not produce TEG traces, eventually leaving a flat line that indicated coagulation failure.

In relation to LY30, we did not observe statistically significant changes at any concentration, consistent with previously published results of in vitro experiments using human blood [38]. The current in vitro study could not entirely replicate the interactions in complicated living organisms; thus, hyperfibrinolysis was excluded [38]. In patients with natural snake envenomation as a result of a snake bite, the common coagulopathy reported using TEG was delayed fibrinolysis [39,40], and LY30 provided information on fibrinolysis before the decrease in fibrinogen concentrations [33]. As such, even though this study did not result in any valid conclusions in relation to LY30 as it focused on detecting early VICC, LY30 can provide useful information about hyperfibrinolysis in clinical practice.

The α-angle displayed a low sensitivity compared with K, MA, and G at the lowest concentration. However, a previous report on pit viper envenomation used TEG to guide additional anti-venom treatment and identified the α-angle as a particularly useful parameter [41]. The α-angle increased several hours before the measurable fibrinogen level in a patient with hypofibrinogenemia [41]. As such, the α-angle is a more valuable parameter for patients with hypofibrinogenemia, where several boluses of anti-venom have already been administered, and a decision about administering additional anti-venom needs to be made.

Fibrinogen concentrations decreased with higher venom concentrations and displayed significant changes at ≥50% LD50_iv_ compared with the control. A possible explanation could be the role of calobin, a thrombin-like enzyme (TLE) present in the venom of *G. ussuriensis* [42], which degrades fibrinogen and causes hypofibrinogenemia [43]. The difference between thrombin and TLE is that TLE mimics thrombin and produces weak fibrin polymerization that gets hydrolyzed by plasmin, unlike thrombin-generated stable polymerization which occurs with the assistance of factor XIII [43]. Accordingly, we detected VICC using fibrinogen; however, its sensitivity to diagnose early VICC was insufficient.

In this study, the lowest venom concentration was 25% LD50_iv_, which is higher than 0.5% LD50_iv_ previously used in a human experiment [38]. The pilot study revealed that the lowest venom concentration tested where TEG showed normal results was 20% LD50_iv_. The difference in venom concentrations between humans and dogs was determined based on a previous study that analyzed the clotting speed among dogs, cats, and humans [6]. For instance, dogs have a higher level of coagulation factors, particularly factor VIII [44], and faster natural plasma clotting time than cats [45]. In addition, domestic dogs have evolved from wolves, i.e., pack-hunters with large prey, such as elks and wild boars, to survive dangerous wounds with a quicker clot time [46]. Rapid clot times denote the faster depletion of coagulation factors, which leads to lower survival rates than those in cats in case of snake envenomation [6]. However, considering the characteristics of the present in vitro experiment and the higher level of coagulation factors in dogs, TEG initiated and detected a normal coagulation process before the depletion of coagulation factors. This resulted in a relatively higher venom concentration (0.5% vs. 25% LD50_iv_) required to simulate coagulopathy, compared with the aforementioned human experiment.

A previous report [47] demonstrated PT and aPTT of shortened time as indications of hypercoagulability and a higher chance of thrombosis. Three dogs exhibited shortened PT at 25% LD50_iv_; however, a PT shorter than the NRL was considered normal in this experiment. Factor hypercoagulability generally produces shorter R values; however, all three dogs had normal R values in the control and 25% LD50_iv_ groups. Thus, we recommend further studies to determine the relationship between shortened PT, aPTT, and TEG.

Snake bites without envenomation are referred to as dry bites [5]. According to a previous study in Sri Lanka, in 86% of the patients admitted to hospital due to snake bites, the bites were diagnosed as dry bites [48]. Dry bites can be attributed to pathophysiologic or non-pathophysiologic conditions. The pathophysiologic conditions include gland infection, ductal calcification or obstruction, venom extraction-related trauma, or defense-related trauma [48]. Contrarily, non-pathophysiologic conditions are related to either an already empty gland or a tactical decision by the snake to preserve its venom during the bite. The aforementioned tactic to spare venom for the next prey or circumstances is termed venom-metering in adult snakes. This is because venom production is associated with metabolic costs and requires considerable time to exert toxic effects [49]. Therefore, anti-venom treatment is not necessary for snake bites confirmed as dry bites, because the injury is analogous to a non-venomous snake bite [50]. However, it is difficult to differentiate between a dry or wet bite, because the bite site may display signs of inflammation and tissue edema [5].

Anti-venom is considered the most effective treatment for snake envenomation, considering it is the only antidote to snake venom. However, the WHO recommends anti-venom only for patients expected to gain benefits that exceed the risk. This can be attributed to the unstable and expensive supply of anti-venom [51]. According to the WHO guidelines, anti-venom is preferably used in envenomated patients with hemostatic abnormalities, cardiovascular problems, abnormal neurologic signs, kidney failure, and worsening local tissue swelling [51]. Moreover, anti-venom can cause adverse reactions, such as anaphylactic shock, pyrogenic reactions, and serum sickness, thus necessitating cautious administration [51]. In addition, performing a skin test before anti-venom administration is controversial because of its extremely low sensitivity and time requirement, which delays anti-venom administration [52].

Under the aforementioned circumstances, distinguishing dry from wet bites or diagnosing early hemostatic abnormalities is the key to treating snake envenomation. TEG produces faster results, appears more suitable than CCTs in detecting early VICC and could be useful in diagnosing and monitoring the patient after anti-venom treatment [38]. We recommend an additional TEG evaluation with CCTs during follow-up for patients admitted to hospital with normal TEG results. Moreover, the unnecessary administration of anti-venom should be suspended. Conversely, clinicians should initiate immediate anti-venom treatment in the event of abnormal TEG results, and in the case of hospitalized patients with hypofibrinogenemia, an abnormal LY30 result would assist the decision making regarding the administration of additional anti-venom boluses. However, the cost of TEG is relatively higher than that of CCTs. This need for expensive infrastructure may limit the applicability of TEG, considering that most of the snake envenomation cases occur in resource-poor environments [12].

This study has a few limitations. First, our in vitro study analyzed the results from a single time point using a small number of dogs, and the results were interpreted based on the NRL values from in vivo studies reported in the literature; therefore, the current findings should be generalized with caution. Second, we could not perform TEG with PlateletMapping^®^ due to financial constraints. An experiment involving platelet inhibition could have provided a better understanding of the venom-induced inhibition of the coagulation factor. In addition, experimentally induced coagulopathy with a stabilization of 30 min may have created a relatively calcium-free environment; therefore, the possibility of coagulopathy caused by low calcium rather than the inhibition of coagulation factor cannot be excluded.

## 5. Conclusions

Through the serial dilution of simulated *G. ussuriensis* venom, we verified concentration-dependent changes in canine blood coagulation parameters. The results were consistent with VICC caused by natural pit viper envenoming. The platelet count did not detect the coagulopathic effects of *G. ussuriensis* venom, unlike TEG and the rest of the CCTs. Nonetheless, TEG effectively identified abnormal coagulation profiles compared with CCTs in in vitro simulated pit viper envenomation. Thus, TEG could be a useful diagnostic strategy for early VICC and preventing treatment delay.

## Figures and Tables

**Table 1:** Sensitivity of classical coagulation tests and TEG parameters: percentages of samples beyond normal reference limits.

Measure	Normal Range	% LD50_iv_ Venom Concentration		
		25%	50%	75%
CCTs				
PT	7.1–8.4 s	0%	30%	50%
aPTT	13.7–25.6 s	10%	50%	70%
Fibrinogen	113–385 mg/dL	0%	50%	70%
PLTs	148–484 K/μL	10%	50%	50%
TEG				
R	1.8–8.6 min	0%	10%	50%
K	1.3–5.7 min	90%	100%	100%
α-angle	36.9–74.6 degrees	30%	70%	90%
MA	42.9–67.9 mm	100%	100%	100%
LY30	NA	NA	NA	NA
G	3.2–9.6 Kdynes/cm^2^	100%	100%	100%

CCTs, classical coagulation tests; PT, prothrombin time; aPTT, activated partial thromboplastin time; PLTs, platelet counts; TEG, thromboelastography; R, reaction time; K, kinetic time; MA, maximum amplitude; LY30, clot lysis at 30 min; G, global strength of the clot; NA, not applicable; s, second; min, minutes.

## Data Availability

The data that support the findings of this study are available from the corresponding author upon reasonable request.

## Supplementary Materials

The following supporting information can be downloaded at: https://www.mdpi.com/article/10.3390/ani12030226/s1, Table S1: Mean values of classical coagulation tests parameters for the control and increasing venom concentrations (% LD50_iv_) with *p*-values and statistical results; Table S2: Mean values of thromboelastography parameters for the control and increasing venom concentrations (% LD50_iv_) with *p*-values and statistical results.

## References

[1] Al-Sadoon M.K., Fahad A.M., Ahamad P.B., Rahman A.A. (2021). Envenomation and the Bite Rate by Venomous Snakes in the Kingdom of Saudi Arabia over the Period (2015–2018). Saudi J. Biol. Sci..

[2] World Health Organization (2019). Snakebite Envenoming: A Strategy for Prevention and Control.

[3] Bigdata Distributed by the Health Insurance Review & Assessment Service in Korea. http://opendata.hira.or.kr/op/opc/olap4thDsInfo.do.

[4] Peterson M.E. (2006). Snake Bite: Pit Vipers. Clin. Tech. Small Anim. Pract..

[5] Pucca M.B., Knudsen C., Oliveira I.S., Rimbault C., Cerni F.A., Wen F.H., Sachett J., Sartim M.A., Laustsen A.H., Monteiro W.M. (2020). Current Knowledge on Snake Dry Bites. Toxins.

[6] Zdenek C.N., Llinas J., Dobson J., Allen L., Dunstan N., Sousa L.F., Moura da Silva A.M., Fry B.G. (2020). Pets in Peril: The Relative Susceptibility of Cats and Dogs to Procoagulant Snake Venoms. Comp. Biochem. Physiol. Part C Toxicol. Pharmacol..

[7] Moon J.M., Koo Y.J., Chun B.J., Park K.H., Cho Y.S., Kim J.C., Lee S.D., Min Y.R., Park H.S. (2020). The Effect of Myocardial Injury on the Clinical Course of Snake Envenomation in South Korea. Clin. Toxicol..

[8] Ramos O.H.P., Selistre-De-Araujo H.S. (2006). Snake Venom Metalloproteases—Structure and Function of Catalytic and Disintegrin Domains. Comp. Biochem. Physiol. C Toxicol. Pharmacol..

[9] Ainsworth S., Slagboom J., Alomran N., Pla D., Alhamdi Y., King S.I., Bolton F.M.S., Gutiérrez J.M., Vonk F.J., Toh C.H. (2018). The Paraspecific Neutralisation of Snake Venom Induced Coagulopathy by Antivenoms. Commun. Biol..

[10] Lim H., Kang H.G., Kim K.H., Goo Kang H., Hwan Kim K., Kang H.G., Kim K.H. (2013). Antivenom for Snake Bite in Korea. J. Korean Med. Assoc..

[11] Debono J., Bos M.H.A., Do M.S., Fry B.G. (2019). Clinical Implications of Coagulotoxic Variations in Mamushi (Viperidae: Gloydius) Snake Venoms. Comp. Biochem. Physiol. Part C Toxicol. Pharmacol..

[12] Park E.J., Choi S., Kim H.H., Jung Y.S. (2020). Novel Treatment Strategy for Patients with Venom-Induced Consumptive Coagulopathy from a Pit Viper Bite. Toxins.

[13] White J. (2005). Snake Venoms and Coagulopathy. Toxicon Off. J. Int. Soc. Toxinol..

[14] Atamna R., Kelmer E., Aroch I., Klainbart S. (2021). *Echis Coloratus* Envenomation in a Dog: Clinical, Hemostatic and Thromboelastometric Findings and Treatment. Clin. Toxicol..

[15] Kopke M.A., Botha W.J. (2020). Thromboelastographic Evaluation of 2 Dogs with Boomslang (*Dispholidus Typus*) Envenomation. J. Vet. Emerg. Crit. Care.

[16] Lieblick B.A., Bergman P.J., Peterson N.W. (2018). Thromboelastographic Evaluation of Dogs Bitten by Rattlesnakes Native to Southern California. Am. J. Vet. Res..

[17] Nagel S.S., Schoeman J.P., Thompson P.N., Wiinberg B., Goddard A. (2014). Hemostatic Analysis of Dogs Naturally Envenomed by the African Puffadder (*Bitis Arietans*) and Snouted Cobra (*Naja Annulifera*). J. Vet. Emerg. Crit. Care.

[18] Olives T.D., Topeff J.M., Willhite L.A., Kubic V.L., Keyler D.E., Cole J.B. (2016). Complete Clinical Course of Envenomation by *Protobothrops Mangshanensis*: Delayed Coagulopathy and Response to *Trimeresurus Albolabris* Antivenom. Clin. Toxicol..

[19] Witham W.R., McNeill C., Patel S. (2015). Rebound Coagulopathy in Patients With Snakebite Presenting With Marked Initial Coagulopathy. Wilderness Environ. Med..

[20] Fry W., Lester C., Etedali N.M., Shaw S., DeLaforcade A., Webster C.R.L. (2017). Thromboelastography in Dogs with Chronic Hepatopathies. J. Vet. Intern. Med..

[21] Park M.S., Martini W.Z., Dubick M.A., Salinas J., Butenas S., Kheirabadi B.S., Pusateri A.E., Vos J.A., Guymon C.H., Wolf S.E. (2009). Thromboelastography as a Better Indicator of Hypercoagulable State after Injury than Prothrombin Time or Activated Partial Thromboplastin Time. J. Trauma Inj. Infect. Crit. Care.

[22] Thakur M., Ahmed A.B. (2012). A Review of Thromboelastography. Int. J. Perioper. Ultrasound Appl. Technol..

[23] Vos J.J., Scheeren T.W.L., Loer S.A., Hoeft A., Wietasch J.K.G. (2016). Do Intravascular Hypo- and Hypervolaemia Result in Changes in Central Blood Volumes?. Br. J. Anaesth..

[24] Morris B.R., DeLaforcade A., Lee J., Palmisano J., Meola D., Rozanski E. (2016). Effects of in Vitro Hemodilution with Crystalloids, Colloids, and Plasma on Canine Whole Blood Coagulation as Determined by Kaolin-Activated Thromboelastography. J. Vet. Emerg. Crit. Care.

[25] Williams P., Yang K., Kershaw G., Wong G., Dunkley S., Kam P.C.A. (2015). The Effects of Haemodilution with Hydroxyethyl Starch 130/0.4 Solution on Coagulation as Assessed by Thromboelastography and Platelet Receptor Function Studies in Vitro. Anaesth. Intensive Care.

[26] Teng C.M., Huang T.F. (1991). Snake Venom Constituents That Affect Platelet Function. Platelets.

[27] Wiinberg B., Kristensen A.T. (2010). Thromboelastography in Veterinary Medicine. Semin. Thromb. Hemost..

[28] Hochleitner G., Sutor K., Levett C., Leyser H., Schlimp C.J., Solomon C. (2017). Revisiting Hartert’s 1962 Calculation of the Physical Constants of Thrombelastography. Clin. Appl. Thromb. Hemost..

[29] Wedasingha S., Isbister G., Silva A. (2020). Bedside Coagulation Tests in Diagnosing Venom-Induced Consumption Coagulopathy in Snakebite. Toxins.

[30] Lee B.J., Hong S., Kim H., Kim T.H., Lee J.H., Kim H., Ryu B., Kim H. (2007). Hematological Features of Coagulopathy and the Efficacy of Antivenin Therapy for a Korean Snakebite. Ann. Surg. Treat. Res..

[31] Pongpit J., Limpawittayakul P., Juntiang J., Akkawat B., Rojnuckarin P. (2012). The Role of Prothrombin Time (PT) in Evaluating Green Pit Viper (Cryptelytrops Sp) Bitten Patients. Trans. R. Soc. Trop. Med. Hyg..

[32] Coggins A., Symes E., Cheeseman C., Salter M. (2019). Thromboelastography for the Detection of Acute Anticoagulant Coagulopathy Associated with Black Snake Envenomation. Emerg. Med. Australas..

[33] Leffers P., Ferreira J., Sollee D., Schauben J. (2018). Thromboelastography in the Management of Snakebiteinduced Coagulopathy: A Case Series and Literature Review. Blood Coagul. Fibrinolysis.

[34] Nag I., Datta S.S., De D., Pal P., Das S.K. (2017). Role of Thromboelastography in the Management of Snake Bite: A Case Report from India. Transfus. Apheresis Sci..

[35] Bolliger D., Görlinger K., Tanaka K.A. (2010). Pathophysiology and Treatment of Coagulopathy in Massive Hemorrhage and Hemodilution. Anesthesiology.

[36] Bolliger D., Seeberger M.D., Tanaka K.A. (2012). Principles and Practice of Thromboelastography in Clinical Coagulation Management and Transfusion Practice. Transfus. Med. Rev..

[37] Chee Y.L. (2014). Coagulation. J. R. Coll. Physicians Edinb..

[38] Fortner G.A., Devlin J.J., McGowan A.J., Boboc M., Natarajan R., Zarow G.J. (2020). Comparison of Thromboelastography versus Conventional Coagulation Tests in Simulated Crotalus Atrox Envenomation Using Human Blood. Toxicon.

[39] Armentano R.A., Bandt C., Schaer M., Pritchett J., Shih A. (2014). Thromboelastographic Evaluation of Hemostatic Function in Dogs Treated for Crotalid Snake Envenomation. J. Vet. Emerg. Crit. Care.

[40] Larréché S., Jean F.X., Benois A., Mayet A., Bousquet A., Vedy S., Clapson P., Dehan C., Rapp C., Kaiser E. (2018). Thromboelastographic Study of the Snakebite-Related Coagulopathy in Djibouti. Blood Coagul. Fibrinolysis.

[41] Rushton W.F., Rivera J.V., Brown J., Kurz M.C., Arnold J. (2020). Utilization of Thromboelastograms in Management of Crotalus Adamanteus Envenomation. Clin. Toxicol..

[42] Cho S.Y., Hahn B.S., Yang K.Y., Kim Y.S. (2001). Purification and Characterization of Calobin II, a Second Type of Thrombin-like Enzyme from Agkistrodon Caliginosus (Korean Viper). Toxicon.

[43] Pradniwat P., Rojnuckarin P. (2014). Snake Venom Thrombin-like Enzymes. J. Toxicol. Toxin Rev..

[44] Giles A., Tinlin S., Greenwood R. (1982). A Canine Model of Hemophilic (Factor VIII:C Deficiency) Bleeding. Blood.

[45] Shea G.M. (1986). Three Western Australian Snake Venoms on Blood Coagulation of the Dog, Cat, Horse and Wallaby. Aust. Vet. J..

[46] Leonard J.A., Wayne R.K., Wheeler J., Valadez R., Guillén S., Vilà C. (2002). Ancient DNA Evidence for Old World Origin of New World Dogs. Science.

[47] Song J., Drobatz K.J., Silverstein D.C. (2016). Retrospective Evaluation of Shortened Prothrombin Time or Activated Partial Thromboplastin Time for the Diagnosis of Hypercoagulability in Dogs: 25 Cases (2006–2011). J. Vet. Emerg. Crit. Care.

[48] Kularatne K., Budagoda S., Maduwage K., Naser K., Kumarasiri R., Kularatne S. (2011). Parallels between Russell’s Viper (Daboia Russelii) and Hump–Nosed Viper (Hypnale Species) Bites in the Central Hills of Sri Lanka amidst the Heavy Burden of Unidentified Snake Bites. Asian Pac. J. Trop. Med..

[49] Hayes W.K., Herbert S.S., Rehling G.C., Gennaro J.F. (2002). Factors That Influence Venom Expenditure in Viperids and Other Snake Species during Predatory and Defensive Contexts. Biology of the Vipers.

[50] Laustsen A.H., María Gutiérrez J., Knudsen C., Johansen K.H., Bermúdez-Méndez E., Cerni F.A., Jürgensen J.A., Ledsgaard L., Martos-Esteban A., Øhlenschlæger M. (2018). Pros and Cons of Different Therapeutic Antibody Formats for Recombinant Antivenom Development. Toxicon.

[51] WHO Regional Office for South-East Asia (2016). Guidelines for the Management of Snakebites.

[52] Shim J.S., Kang H., Cho Y., Shin H., Lee H. (2020). Adverse Reactions after Administration of Antivenom in Korea. Toxins.

