# Supplementary material for: Hemostatic Analysis of Simulated Gloydius ussuriensis Envenomation Using Canine Blood: A Comparison of Thromboelastography and Classical Coagulation Tests
Source: Animals (Basel). 2022 Jan 18;12(3):226. doi: 10.3390/ani12030226 (PMC8833665; doi:10.3390/ani12030226)
Supplement: Supplementary file 1 [file animals-12-00226-s001.zip › animals-1519767-supplementary.pdf]

Table S1. Mean values of classical coagulation tests parameters for the control and increasing venom concentrations (%$LD50_{iv}$) with *p*-values and statistical results.

CCTs	Normal Range	Mean	SD	*n*	Normality	*p*-Value
**PT**	7.1–8.4 s					<0.001
Control		7.47	0.33	10	Y	
25%		7.4	0.36	10	N	0.691
50%		7.61	0.53	10	Y	0.013
75%		14.01	10.24	10	N	<0.001
**aPTT**	13.7–25.6 s					<0.001
Control		20.79	2.519	10	Y	
25%		20.85	4.488	10	Y	0.802
50%		24.47	5.567	10	Y	<0.001
75%		65.4	81.58	10	N	<0.001
**Fibrinogen**	113–385 mg/dL					0.004
Control		189.4	53.51	10	Y	
25%		196.9	57.41	10	Y	0.275
50%		124.1	86.67	10	N	0.159
75%		112.2	125.1	10	N	0.003
**PLTs**	148–484 K/μL					0.2
Control		188.8	26.36	10	Y	
25%		182.6	46.4	10	Y	0.377
50%		156.1	51.45	10	Y	0.07
75%		154.1	36.63	10	Y	0.07

CCTs, classical coagulation tests; SD, standard deviation; PT, prothrombin time; aPTT, activated partial thromboplastin time; PLTs, platelet counts; s, seconds

Table S2. Mean values of thromboelastography parameters for the control and increasing venom concentrations (%$LD50_{iv}$) with *p*-values and statistical results.

TEG	Normal Range	Mean	SD	*n*	Normality	*p*-Value
**R**	1.8–8.6 min					0.187
Control		3.97	1.14	10	Y	
25%		4.09	1.09	10	Y	0.93
50%		5.2	2.44	10	Y	0.72
75%		9.44	6.67	10	N	0.068
**K**	1.3–5.7 min					<0.001
Control		2.84	0.82	10	Y	
25%		8.38	4.11	10	N	<0.001
50%		16.82	9.34	10	N	<0.001
75%		25.99	8.47	10	N	<0.001
**α-angle**	36.9–74.6 degrees					<0.001
Control		55.55	8.57	10	Y	
25%		39.85	9.52	10	Y	0.001
50%		31.01	11.52	10	Y	<0.001
75%		16.24	14.01	10	Y	<0.001
**MA**	42.9–67.9 mm					<0.001
Control		55.92	5.58	10	Y	
25%		32.84	7.05	10	Y	<0.001
50%		25.23	7.59	10	Y	<0.001
75%		12.09	2.4	10	Y	<0.001
**LY30**	NA					NA
Control		0.11	0.2	10	N	
25%		0.02	0.06	10	N	NA
50%		0.83	2.5	9	N	NA
75%		1.67	5.28	10	N	NA
**G**	3.8–10.6 dynes/cm^2					<0.001
Control		6.51	1.6	10	Y	
25%		2.51	0.76	10	Y	<0.001
50%		1.8	0.69	10	Y	<0.001
75%		0.76	0.67	10	N	<0.001

Notably, one value of LY30 was missing due to an abrupt halt in TEG during the analysis for an unknown reason. TEG, thromboelastography; SD, standard deviation; R, reaction time; K, kinetic time; MA, maximum amplitude; LY30, clot lysis at 30 min; G, global strength of the clot; NA, not applicable; min, minutes
